# Rationale and Design of a Randomised Proof-of-Concept Trial to Assess the Safety of Early Discharge Using Index Microcirculatory Resistance in Patients with Acute Myocardial Infarction: SECURE Study

**DOI:** 10.3390/jpm16040207

**Published:** 2026-04-07

**Authors:** Muntaser Omari, Mohamed Ali, Luke Spray, Adam McDiarmid, Mohammad Alkhalil

**Affiliations:** 1 Cardiothoracic Centre, Freeman Hospital, Newcastle-upon-Tyne NE7 7DN, UK; 2 Translational and Clinical Research Institute, Newcastle University, Newcastle-upon-Tyne NE2 4HH, UK

**Keywords:** STEMI, early discharge, left ventricle, IMR, MRI

## Abstract

**Background**: Current guidelines acknowledge that early discharge is not associated with late mortality and that in-hospital length of stay (LOS) of 48–72 h should be considered following successful primary percutaneous coronary intervention (PPCI) in low-risk patients. Recent studies have highlighted the safety of very early discharge after PPCI in highly selected low-risk patients; however, objective tools to guide discharge timing remain limited. The Index of Microcirculatory Resistance (IMR) offers a quantitative assessment of microvascular function and may help identify patients suitable for very early discharge. We aimed to evaluate the feasibility of using IMR to guide very early discharge in patients who underwent uncomplicated PPCI. **Study design and objectives**: The Safety of Early Discharge Using Index Microcirculatory Resistance in Patients with Acute Myocardial Infarction (SECURE) study is designed to assess the feasibility of using IMR, measured immediately following successful PPCI, to guide early discharge from hospital within 24 h. The SECURE study is a prospective, proof-of-concept, functional non-inferiority, single-centre, randomised, open-label trial to determine if patients with low IMR can be safely discharged when compared to standard discharge policy. The SECURE study will recruit 82 patients with low IMR following successful PPCI. Participants will be 1:1 randomised to either standard discharge timing or very early discharge (within 24 h). The left ventricle ejection fraction will be assessed using cardiac magnetic resonance imaging. A telephone follow-up at 3 months will be arranged. Clinical events are collected as secondary and exploratory safety endpoints. **Conclusions**: The SECURE study will provide proof-of-concept data about the feasibility of using IMR to guide very early discharge following PPCI. If successful, this study will provide data to plan for a larger study to determine the safety of this personalised approach.

## 1. Introduction

Primary percutaneous coronary intervention (PPCI) is the standard treatment option for patients presenting with ST-elevation myocardial infarction (STEMI) [1]. Successful reperfusion therapy using PPCI has resulted in a significant decrease in mortality and a progressive reduction in hospital length of stay (LOS) [2]. As outcomes have improved, earlier hospital discharge has become a priority for both patients and healthcare systems. This was reflected in current guidelines, acknowledging that early discharge is not associated with late mortality and in-hospital length of stay (LOS) of 48–72 h for patients who are considered low risk following successful PPCI should be considered [3]. Nonetheless, objective tools that can define low-risk patients following successful PPCI are currently lacking.

IMR is a quantitative measure that can directly evaluate the status of the myocardium immediately after PPCI. It has the potential to identify patients who are at low risk and may be suitable for early discharge following STEMI presentation. IMR has an important predictive role in patients undergoing PPCI. It correlated with the size of the infarct when measured using peak creatinine kinase, echocardiographic wall motion score, regional 18F-fluorodeoxyglucose (FDG) uptake imaged by positron emission tomography, and infarct volume on cardiac magnetic resonance (CMR) imaging [4,5,6]. Moreover, IMR immediately at the end of PPCI was identified as a reliable predictor of early adverse clinical outcomes [7]. Therefore, IMR may be used as a stratification tool to identify low-risk patients who may be suitable for a ‘step-down’ bed immediately following PPCI, rather than the coronary care unit. This group could also be targeted for an early discharge strategy.

Notably, STEMI patients are kept in hospital to monitor and manage any potential life-threatening complications, as well as to optimise medical therapy and to educate patients on a healthy lifestyle [1,3]. An approach of very early hospital discharge may limit the opportunities to maximise medical treatment, compromising left ventricle recovery following STEMI presentation. Therefore, a comparison of left ventricle function according to hospital discharge strategies would pertain to the role of optimising medical treatment in this setting.

## 2. Rationale of the SECURE Study

Identifying low-risk patients presenting with STEMI who underwent successful PPCI and are suitable for early hospital discharge would be beneficial for patients, physicians, and health care providers. It is well-established that patients feel more supported at home and are often more satisfied with short in-hospital LOS, particularly when discharge planning is well-structured and includes short-term follow-up. Similarly, early hospital discharge will increase efficiency and optimise the use of health care resources, including treating more patients within the same bed capacity.

Determining low-risk STEMI patients who are candidates for early hospital discharge remains to be established. IMR is a reproducible and safe measurement that can be calculated using the guidewire-based thermodilution method, which is widely available in coronary catheterisation laboratories [8]. IMR assessment can be performed immediately following PPCI and can provide prognostic information for patients presenting with AMI [9,10]. Therefore, we set out to study the feasibility of using IMR in patients presenting with STEMI who underwent successful PPCI to guide early hospital discharge. A comparison of patients without major microvascular dysfunction, defined as IMR of equal or less than 40 units, is planned according to their hospital discharge strategies. This means comparing patients with low IMR who are assigned to early hospital discharge, i.e., within 24 h, versus standard in-hospital LOS. We expect that patients with early hospital discharge would have similar left ventricular function on CMR at three months, without increased risk of major adverse clinical events, including death, recurrent myocardial infarction, unplanned revascularisation, readmission to hospital with heart failure, or cardiac arrest, sustained ventricular tachycardia/fibrillation, and cardioverter defibrillator implantation.

## 3. Methods

### 3.1. Study Design

The SECURE study is an investigator-initiated, proof-of-concept, functional, non-inferiority, single-centre, randomised open-label study (ISRCTN56383403, registration date on 5 July 2024). Patients over the age of 18 but less than 90 who underwent successful PPCI with preserved microvascular function and meet the inclusion criteria will be 1:1 randomised to either early hospital discharge, i.e., within 24 h or standard in-hospital LOS. The study will also include a parallel observational registry cohort consisting of non-randomised patients with elevated IMR (>40).

**Figure 1:**
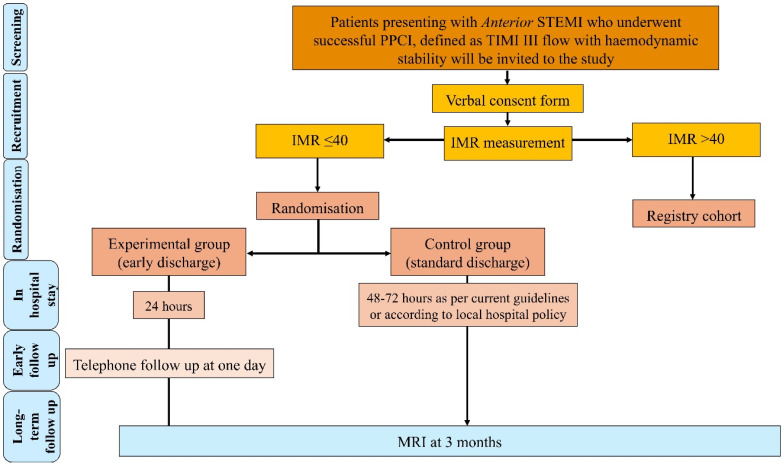
Study flow chart.

Each participant is expected to remain in the study for 12 months, during which a telephone follow-up is planned at 3 months as well as 12 months. Recruitment is expected to be completed in 24 months with the completion of the study when the last patient undergoes their final clinic follow-up. The study will be subject to an extension should the target study sample size not be achieved by the pre-specified end date to maintain the scientific integrity of the study (Figure 1).

The SECURE study will aim to recruit STEMI patients with (1) large calibre culprit artery of at least 3 mm in diameter, (2) successful angiographic results (Thrombolysis in Myocardial Infarction (TIMI) flow of 3) (irrespective of TIMI flow at presentation and patients with occluded vessel can also be included in the study), and (3) preserved microvascular function identified as IMR ≤ 40. The rationale behind including patients with only a large culprit vessel diameter is to reduce the probability of including patients with relatively small infarcts that can mask any unwarranted effect of early hospital discharge on left ventricle function. This group is likely to have normal left ventricle function and can therefore dilute the treatment effect of optimising medical therapy according to the duration of in-hospital LOS.

### 3.2. Study Setting

The SECURE study is a single-centre study recruiting patients presenting with STEMI who are transferred on the PPCI pathway at the Freeman Hospital in Newcastle upon Tyne.

### 3.3. Study Participants

Patients presenting with STEMI secondary to a culprit vessel of at least 3.0 mm in diameter and undergoing PPCI will be screened for enrolment. Patients need to fulfil the inclusion and exclusion criteria prior to randomisation (Table 1). Following assessment of microvascular function, patients will be 1:1 randomised to either early hospital discharge (within 24 h, experimental group) or standard in-hospital LOS (control group).

### 3.4. Study Procedures

#### 3.4.1. Screening and Assent

Given the emergency setting of the SECURE study, obtaining full informed consent from potential participants prior to any study procedure would not be feasible. Therefore, upon completing the PPCI and after reviewing the inclusion and exclusion criteria, a member of the direct care team will approach the patient whilst they remain inside the catheterisation laboratory. This member will be fully trained in the study aims and its inclusion criteria. After explaining the study and its design, including the risks and benefits of participating in the study, a verbal assent will be obtained after ensuring the patient is clinically stable and judged to have the capacity to understand the study. Importantly, the patient will be informed that there is a chance that they may not meet the inclusion criteria if their microvascular function is deemed impaired according to the IMR assessment. Participants retain the right to withdraw consent, and data will be excluded if full consent is declined. To ensure generalisability of the study, a flow diagram detailing the number of screened and randomised participants will be recorded.

#### 3.4.2. Microvascular Function Assessment

**Figure 2:**
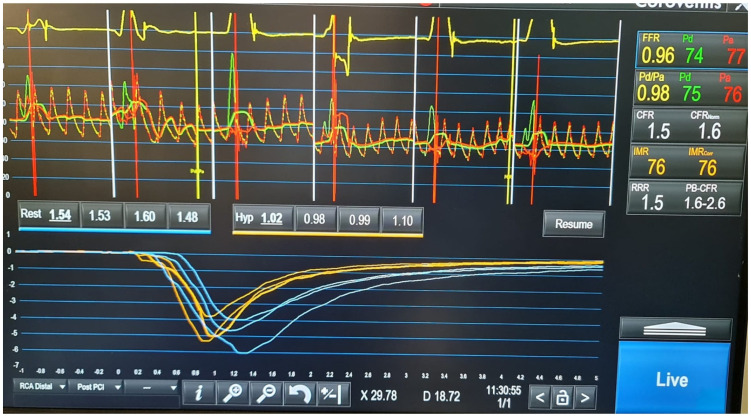
An illustrative example of coronary microcirculation assessment using PressureWire™ X. The top row represents a patient with preserved microcirculation function (IMR less than 40), and the bottom row illustrates a patient with impaired microcirculation assessment (IMR above 40). Blue curves show resting transit times and orange shows hyperaemic transit times. Resting as well as hyperaemic pressure and flow indices are displayed on the right side of the Figure.

Upon assent, a full coronary microcirculation assessment will be performed using guidewire-based technology (PressureWire™ X Guidewire, Abbott, IL, USA) and the thermodilution method in the culprit vessel, as previously described [11,12,13]. Following an intra-coronary bolus of 200–300 µg of isosorbide dinitrate, the guidewire will be calibrated and equalised before advancing toward the third segment of the infarct-related artery. Baseline parameters of microvascular function will be recorded, including mean aortic pressure (Pa), mean distal pressure (Pd), and mean transit time (mTt). The latter will be calculated as the average transit time measurement from three separate intracoronary injections of 3 mL of room temperature 0.9% saline solution. Hyperaemia will be induced by administering an intravenous infusion of adenosine at a rate of 180 µg/kg/min for at least two minutes. A repeat measurement of Pa and Pd will be recorded, as well as mTt at stress. This will be calculated using three separate intra-coronary injections of 3 mL of room temperature 0.9% saline solution. Fractional flow reserve (FFR), coronary flow reserve (CFR), and IMR using the current equations and reported instantaneously by the site study team (Figure 2):

FFR = Pd/Pa (during hyperemia)

CFR = mTt_baseline_/mTt_hyperemia_

IMR = Pd × mTt (during hyperemia)

#### 3.4.3. Randomisation

The SECURE study will only randomise participants who have preserved microvascular function as defined by IMR ≤ 40. Based on previous studies [7,14], we expect that two-thirds of patients with good procedural outcomes, including TIMI 3 flow, will have preserved microvascular function as defined by the SECURE study methodology.

Once microvascular function assessment is performed, patients with IMR ≤ 40 will be 1:1 randomised by means of a web-based randomisation secure method with no stratification block. Blinding or masking of the randomisation group cannot be applied, given the nature of the study. Therefore, patients as well as physicians will be aware of the discharge strategy; however, the analysis of the primary endpoint of left ventricular function will be blindly performed.

#### 3.4.4. Full Informed Consent and Baseline Data Collection

Following the completion of the PPCI, full written consent will be sought from the patient. Given the nature of the study and the randomisation process, it will be recommended to approach the patient as soon as practical to do so. This will allow the patient to read, reflect and ask any questions related to the study and its procedure.

Upon obtaining a written consent form, baseline clinical data will be recorded. This will include medical history, including the patient’s demographic, procedural and medication details. Routine laboratory blood results, including cardiac troponin level and 12-lead electrocardiogram, will also be recorded.

Patients will undergo standard in-hospital management, including cardiac monitoring, echocardiography, optimisation of medical therapy, and ongoing education.

#### 3.4.5. Clinical Follow-Up

A 24 h telephone clinic follow-up will be scheduled for patients with low IMR (≤40) who are randomised into the early discharge group. Patients in this group will be contacted the following day to ensure well-being and ongoing education regarding diagnosis and future management. This visit will also be used as an opportunity to optimise medical therapy. Introducing medications following STEMI presentation is not universally standardised, and titration does not follow a specific protocol; therefore, this will be decided by the same study team to minimise confounders and ensure consistency in the management of their medications. It will also be used to ensure that the patient has a good understanding of the current medical treatment, including antiplatelet, lipid-lowering, and other secondary prevention therapies.

At 3 months, patients with low IMR (≤40), irrespective of their discharge strategies, will have a scheduled telephone follow-up to collect data on any medical treatment or clinical adverse events. This will include cardiac and cerebrovascular events, including cardiac and all-cause death. Data on any hospital admission for cardiac or non-cardiac reasons will also be recorded. A full medication list will also be recorded, including doses of cardiovascular secondary preventive therapies. A comparison of medication doses according to the hospital discharge strategy will be performed by standardising current doses of each of the guidelines-recommended preventive treatments. This will be executed by calculating the ratio of current to the maximal dose of each therapy and, subsequently, averaging the results to reflect the current treatment that each patient is receiving at three months. This value will be referred to as the average standardised medical treatment (ASMT) dose.

A second telephone follow-up will be scheduled at 12 months to obtain similar data from patients with low IMR who are assigned to either early discharge or standard in-hospital LOS.

#### 3.4.6. Cardiac Magnetic Resonance Imaging

All patients in the randomised cohort (both experimental and control groups) will be invited to undergo CMR at three months. Standard MRI sequences include real-time cine imaging, and late gadolinium enhancement will be performed. This visit will enable assessment of left ventricle function (primary endpoint), left ventricle volumes, and infarct size (secondary endpoints).

### 3.5. Objectives and Outcome Measures

The study’s objectives and outcome measures are presented in Table 2. The primary objective of the SECURE study is to assess whether an early hospital discharge strategy, i.e., within 24 h, would have any impact on left ventricle recovery as assessed on CMR. This will be evaluated in patients with preserved microvascular function, defined by low IMR, following successful PPCI. One rationale for keeping STEMI patients in the hospital is to optimise medical treatment and emphasise education about the current condition. Therefore, by adopting early hospital discharge, it is plausible that opportunities to titrate medical treatment and ensure compliance may be compromised. This may result in smaller drug doses and subsequently lower left ventricular function that can be detected using CMR.

The secondary objectives of the SECURE study will include providing clinical safety data on using coronary physiology to guide early hospital discharge. Clinical events are collected as secondary and exploratory safety endpoints, and the study is not powered to detect any difference between the two randomised groups. Data on all-cause mortality, recurrent myocardial infarction, unplanned revascularisation, readmission with heart failure, cardiac arrest, sustained ventricular tachycardia/fibrillation, and cardioverter defibrillator implantation will be collected at 3 months as well as 12 months. This is to ensure that the early discharge strategy is not associated with increased clinical adverse events that may be negated by in-hospital optimisation of medical treatment. Additionally, the SECURE study will assess the cost-effectiveness of using coronary physiology assessment for the National Health Service (NHS) to identify a group of patients who are suitable for early hospital discharge. This remains an exploratory analysis, but it will provide important data to assess the health economic benefits from implementing an early discharge strategy in the low-risk population. The intended analysis will evaluate whether any potential cost-saving, by reducing the number of days in coronary care units and cardiology wards, is not offset by using additional guidewires to assess microvascular function or by setting up a nurse-led follow-up clinic following hospital discharge. Importantly, it is recognised that early outpatient follow-up may reduce readmission rate, which was reported to be more than 10% in patients presenting with acute MI [15]. Finally, the SECURE study will evaluate the functional status and severity of angina symptoms according to the hospital discharge strategy.

### 3.6. Statistical Analysis

The obtained data will be assessed for normality distribution and reported as means (and standard deviation) or medians (and inter-quartile range) accordingly. The primary endpoint of left ventricle ejection fraction will be compared according to the in-hospital LOS groups using an unpaired *t-test* (or Mann–Whitney U test if appropriate). Other cardiac MRI endpoints, such as left ventricle volume and infarct size, will be evaluated using standard statistical tests depending on whether the data will be normally distributed or skewed. The composite clinical endpoints will be assessed with the use of cumulative incidence curves and the estimated Cox proportional hazards model. Each component of the clinical endpoints will be similarly compared between the two groups using chi-square or Fisher’s exact test, as appropriate.

The study is designed to establish the non-inferiority of early discharge compared to standard practice on left ventricle function at three months following STEMI presentation in patients with preserved microvascular function. Previous data suggested that left ventricle ejection fraction was 46 ± 8% in patients presenting with STEMI who underwent an uncomplicated procedure with final TIMI 3 flow and were hemodynamically stable post PPCI [16,17]. Assuming a non-inferiority margin of 5% difference in left ventricle ejection fraction with 80% power at an alpha level of 0.025, a sample size of 82 patients is required (41 patients in each group). An absolute difference of 5% in LVEF was selected based on prior studies, whereas differences in this magnitude are generally considered within the range of measurement variability and unlikely to translate into meaningful differences in clinical outcomes, particularly in haemodynamically stable, low-risk patients [16,18]. Given that 25% of patients presenting with STEMI and TIMI 3 flow will have IMR > 40 [19], and assuming that 5% of included patients will be lost to follow-up, the total sample size is estimated as 122 patients. The primary analysis will be conducted according to the intention-to-treat principle, but per-protocol analysis will also be performed. Missing CMR data will be handled using predefined sensitivity analyses.

### 3.7. Ethics and Regulatory Considerations

The investigators will ensure that the SECURE study will be conducted in accordance with relevant regulations, Good Clinical Practice, and the principles of the Declaration of Helsinki. The protocol and all patient-facing documents have been submitted and received approval from the South East Scotland Research Ethics Committee 01 (reference number 23/SS/0116). The investigators will submit and, where necessary, obtain approval for all substantial amendments to the original approved documents.

Direct access will be granted to authorised representatives from the Sponsor or host institution for monitoring and/or audit of the study to ensure compliance with regulations. Any audit or monitoring visit will be done in accordance with the current approved protocol, GCP, relevant regulations and standard operating procedures.

## 4. Discussion

The SECURE study will be the first study to apply the use of coronary microcirculation assessment to identify STEMI patients who may be candidates for early hospital discharge, i.e., within 24 h. This study will aim to address a major gap in contemporary PPCI care related to the absence of an objective approach to guide decisions regarding in-hospital LOS. Current guidelines acknowledge that short in-hospital LOS of less than 72 h is not associated with adverse events in low-risk patients following successful PPCI [3].

Several risk models were proposed to identify candidates for early discharge, including the Second Primary Angioplasty in Myocardial Infarction (PAMI-II) criteria, and the Zwolle primary PCI Index [1,20,21]. However, those risk models were derived using data from more than two decades ago, and contemporary PPCI has significantly evolved with better triage to minimise delay to the catheterisation laboratory, as well as improvement in pharmacological and non-pharmacological treatments, including stent designs. More recent studies reported the safety of very early discharge of less than 48 h in patients presenting with STEMI and undergoing successful PPCI [17,22]. Nonetheless, these studies relied on certain clinical and angiographic markers, such as infarct location, presence of multi-vessel disease, and multi-vessel PCI, to aid decision-making regarding early discharge [17,22]. Despite being considered low risk, a significant proportion of patients stayed in-hospital for more than 48 h, highlighting a potential selection bias and the need to use a more reliable metric to identify patients who are candidates for early hospital discharge [17,22]. Moreover, such a metric needs to reflect the pathophysiological process and can characterise the injured myocardium (and microvascular function) to safely guide early hospital discharge.

IMR provides a robust measure of microvascular integrity and has shown excellent ability in predicting the recovery of left ventricle function [5]. It has also been linked to clinical outcomes, and major clinical adverse events were significantly higher in patients with IMR > 40 compared to those with IMR ≤ 40 (17.1% vs. 6.6%, *p* = 0.027) [10]. In fact, IMR > 40 was associated with more than a 4-fold increase in risk of death and heart failure at one year [7,23]. Importantly, IMR exists as a continuum, and a pooled analysis of 1265 patients from 6 cohorts highlighted that serious adverse clinical events such as cardiac death can be predicted using a higher IMR cutoff (above 70) in patients presenting with STEMI [24]. Nonetheless, the same study highlighted that IMR above 40 was associated with more cardiac death, a higher composite of cardiac death and heart failure, a larger infarct size and more frequent microvascular obstruction and intra-myocardial haemorrhage [24]. Whilst dichotomisation may reduce granularity, the selected binary threshold was chosen to enhance clinical interpretability and real-world applicability of the discharge strategy.

The randomisation process within the SECURE study is planned without any stratification. An imbalance between treatment groups may be prevented using stratification for known factors that influence prognosis [25]. There are several clinical and angiographic features that are considered key prognostic variables in patients presenting with STEMI; however, their interaction with the short length of stay is yet to be determined. Overall, IMR may serve as a useful tool to identify patients who may be at low risk of early adverse events and potentially suitable for early discharge from the hospital.

In a previous study that included 261 patients who underwent PPCI, the incidence of cardiac complications before hospital discharge or within 30 days, defined as the composite of cardiac death, cardiogenic shock, pulmonary oedema, malignant arrhythmias, cardiac rupture, and presence of left ventricular thrombus, was 8.4% (22 out of 261). IMR ≤ 40 was recorded in all patients without in-hospital complications [7]. On the other hand, 1 in 7 patients with IMR > 40 was associated with in-hospital adverse events [7]. It had a sensitivity of 100% with a negative predictive value of 100% and outperformed existing criteria for early discharge, such as PAM-II and the Zwolle scores [7].

By incorporating IMR into discharge decision-making, clinicians may be able to identify patients at low risk of complications while reducing unnecessary hospitalisation.

If early discharge guided by IMR proves non-inferior in terms of LV functional recovery—and safe, acceptable to patients, and cost-effective—the SECURE strategy could transform post-STEMI pathways, reduce bed occupancy, and optimise resource allocation across healthcare systems. Importantly, the SECURE study targets selected low-risk patients presenting with STEMI who underwent a successful procedure without hemodynamic instability. The enrolment of this narrow STEMI subgroup would not enable physicians to apply the study’s findings to a broader population and would limit its generalisability. Implementing an IMR-guided early discharge strategy requires a larger study to ensure the safety of this approach.

In conclusion, the SECURE study will provide proof-of-concept data about the feasibility of using IMR to guide very early discharge following PPCI. If successful, this study will provide data to plan for a larger study to determine the safety of this personalised approach.

## Figures and Tables

**Table 1:** Inclusion and exclusion criteria of the SECURE study.

Inclusion criteria
Age 18–90 years
Successful revascularisation with TIMI 3 flow
Haemodynamic stability following PPCI Preserved microvascular function, defined as IMR ≤ 40
Ability to provide verbal consent
Exclusion criteria
Out-of-hospital cardiac arrest
Suboptimal angiographic results
Previous coronary artery bypass grafting
Cardiogenic shock or pulmonary oedema
Planned staged PCI during index admission
Known contraindication to adenosine
Known contraindication for CMR
Pregnancy or breastfeeding

**Table 2:** Primary and secondary objectives of the SECURE study.

Objectives	Outcome Measures	Evaluation Timepoints
Primary Objective Impact of early discharge on left ventricle function in STEMI patients with preserved microvascular function	Left ventricle ejection fraction as estimated on CMR	3 months
Secondary Objectives (a) Safety of early discharge strategy	The composite of all-cause mortality, recurrent myocardial infarction, unplanned revascularisation, readmission with heart failure, cardiac arrest, sustained ventricular tachycardia/fibrillation, and cardioverter defibrillator implantation.	3 and 12 months.
(b) Cost-effectiveness	The potential cost-saving of using coronary physiology to guide early hospital discharge in patients presenting with STEMI and preserved microvascular function.	3 months
(c) Impact on functional status	The severity of angina symptoms and functional status in STEMI patients with preserved microvascular function according to their hospital discharge policy	3 months

## Data Availability

The raw data supporting the conclusions of this article will be made available by the authors on request.
